# Supporting Vaccination on TikTok During the COVID-19 Pandemic: Vaccine Beliefs, Emotions, and Comments

**DOI:** 10.3389/fpsyg.2022.938377

**Published:** 2022-07-07

**Authors:** Xiaopei Wang, Renyi He

**Affiliations:** ^1^School of Languages and Communication Studies, Beijing Jiaotong University, Beijing, China; ^2^School of Journalism and Communication, The Chinese University of Hong Kong, Shatin, Hong Kong SAR, China

**Keywords:** 5C model, vaccine hesitancy, COVID-19, emotion, TikTok

## Abstract

TikTok has been one of the most important social media platforms where pandemic-related information converged and has been disseminated. However, how vaccination-related visual content, particularly pro-vaccine videos, influences audiences remains unclear. Using Betsch et al.’s 5C model and Ekman’s basic emotion model, we identified 200 trending videos under the hashtag #vaccine on TikTok, and examined the types of vaccine-related beliefs and emotions expressed in videos and the relationship between beliefs, emotions, and supportive comments. Confidence and joy were the most frequently expressed belief and emotion, respectively; confidence (*B* = 14.84, *P* < 0.05), surprise (*B* = 11.29, *P* < 0.05), and sadness (*B* = 37.49, *P* < 0.01) predicted the number of supportive comments. This study expands the 5C framework of vaccine hesitancy into the analysis of pro-vaccine content on social media and offers detailed insights into the specific type of beliefs and emotions and their effects. Practical implications regarding how to address vaccine hesitancy are discussed.

## Introduction

The COVID-19 pandemic has produced an unprecedented global public health crisis. Vaccination is believed to be an effective method to control the contagion, reduce mortality, and strengthen the public health system (Committee on Equitable Allocation of Vaccine for the Novel Coronavirus et al., 2020). Despite the availability of vaccination services, the number of Americans reporting that they would get vaccinated has fluctuated (Funk and Gramlich, 2021). By undermining people’s response, the “infodemic” and misinformation on social media has contributed to vaccine hesitancy (World Health Organization, 2020), leading to unreported preventable infections (Loomba et al., 2021; Solís Arce et al., 2021).

Vaccine hesitancy refers to vaccine uptaking behaviors that present as a “delay in acceptance or refusal of vaccination despite availability of vaccination services” (MacDonald, 2015). It also increases the likelihood of accepting misleading claims (MacFarlane et al., 2020), resisting scientific evidence, or making associated poor health choices (van der Linden, 2015). Based on the theory of reasoned action (TRA), studies have found that vaccine uptaking behaviors can be predicted by individuals’ intentions (e.g., Wu et al., 2020; Akther and Nur, 2022). Further, existing analyses have shown that negative attitudes toward the COVID-19 vaccine are associated with a refusal to accept it (Taylor et al., 2020; Wilson and Wiysonge, 2020), while positive attitudes toward it increase its acceptance rates (Akther and Nur, 2022). Therefore, users’ attitude is also an important predictor of vaccine hesitancy.

Considering that beliefs and emotions influence people’s attitudes to illness (Diefenbach and Leventhal, 1996), we believe that vaccine-related beliefs and emotions could influence vaccine hesitancy through changing people’s attitudes. Although many previous studies have focused on the influence of anti-vaccine beliefs and emotions on attitudes toward vaccines (Venkatraman et al., 2015; Pivetti et al., 2021a,b), the influences of specific types of beliefs and emotions on attitudes toward vaccination remain unclear. However, it is important to analyze such influence as research shows that negative beliefs with negative emotions can elicit negative attitudes (Bean, 2011; Kata, 2012).

The role of emerging social media in overcoming vaccine hesitancy is also an underexplored area. Particularly, TikTok has been among the most important platforms on which pandemic-related information has been disseminated and exchanged, with 77% of Americans regularly turning to TikTok to keep up with COVID-19 vaccine news (Mitchell and Liedke, 2021). Compared with confronting anti-vaccine beliefs directly, it is believed that a more effective way of doing so is to empower vaccination supporters to promote their positive experiences online (Chadwick et al., 2021). However, how to convince them remains a challenge amid anti-vaccine information. To date, existing research has not examined how pro-vaccine content is portrayed on social media, not to mention its effects. As TikTok has been implementing measures to curb the spread of misinformation since December 2020 (Manjunath, 2020), most of the top-ranking videos on the platform are pro-vaccine, which made it an ideal platform for studying the pro-vaccine content and its effects. Therefore, this study investigates beliefs and emotions expressed in pro-vaccine content on TikTok and explores their effects on users’ attitudes toward vaccines. The findings offer new insights into overcoming vaccine hesitancy by elucidating the relationship between beliefs, emotions, and user reactions, and inform the design of better strategies to promote vaccination as well as combat misinformation.

## Beliefs and Emotions Embedded in the Vaccination-Related Content on Social Media

The relationship between beliefs about vaccines and treatment intentions has become a heated topic of academic interest (Bynum et al., 2012; Mitra et al., 2016; Karamanidou and Dimopoulos, 2018). Scholars have found that the spread of anti-vaccine beliefs about the consequences of vaccination has led to greater vaccine and treatment hesitancy (Bogart et al., 2021; Pivetti et al., 2021a,b), and consequently, caused individuals to delay or refuse to be immunized (Freeman et al., 2020). To better understand and analyze vaccination beliefs and subsequent behaviors, the Strategic Advisory Group of Experts on Immunization of the World Health Organization put forward the “3C model,” identifying complacency, convenience, and confidence as three factors that explain the psychological process of vaccine hesitancy (Larson et al., 2014). Under the framework of the 3C model, scholars have developed different theoretical models to explain the determinants of vaccine uptake decisions (Thomson et al., 2016). One of the more notable models is the 5C model (Betsch et al., 2018), which consists of five dimensions: confidence, constraints, complacency, calculation, and collective responsibility. Prior research with the 3C and 5C models have established their validity (Betsch et al., 2018; Kwok et al., 2021). These models provide an effective framework for analyzing pro-vaccine contents. Therefore, the first research question (RQ) of this study is as follows:

RQ1: What kind of vaccine beliefs (confidence, constraints, complacency, calculation, and collective responsibility) are expressed in pro-vaccine videos on TikTok?

Emotions play a decisive role in information dissemination on social media (Lu and Gall Myrick, 2016). It tends to be closely related to the gratifications of entertainment and sensationalism, and is deemed to improve the delivery of information and capture audiences’ attention (Pantti, 2010). People display strong but diverse emotions during discussions of vaccination (Covolo et al., 2017; Moon and Lee, 2020; Bogart et al., 2021; Lyu et al., 2021; Yin et al., 2021). Those who oppose vaccination were more likely to use emotional language on social media during the pandemic (Germani and Biller-Andorno, 2021); for instance, expressing anger (Mitra et al., 2016). However, it is equally important to understand how vaccination supporters strategically expressed different types of emotions to have their voices heard and counter anti-vaccine arguments (Chou and Budenz, 2020).

Most COVID-19-related videos on TikTok at the beginning of the pandemic featured health-related content that evoked humor or parody (Southwick et al., 2021). Later content analysis showed that the most frequently expressed emotion was hope (Li et al., 2021). However, parody or humor should be regarded more as a technique of attracting attention (Tolentino, 2019) rather than an emotion regarding the COVID-19 vaccination. Hope is more related to social media content concerning the overall situation of the pandemic, rather than vaccination. Therefore, we examined the emotions expressed about COVID-19 vaccination on TikTok, and proposed the second RQ:

RQ2: What emotions are expressed in COVID-19 vaccine-related videos on TikTok?

Psychological theories suggest that emotions are closely associated with the human cognitive system (Cannon, 1927). Previous empirical studies have shown how beliefs and emotions are linked. For instance, anger mediated the relationship between misinformation and attitude toward getting vaccinated (Featherstone and Zhang, 2020). Existing studies have distinguished between pro-/anti-vaccine beliefs and positive/negative emotions but have neglected the diverse relationship between beliefs and emotions. Thus, a detailed correlation between vaccine beliefs and emotions should be investigated, to enhance our understanding of the visual content related to vaccination. The third RQ is as follows:

RQ3: What is the relationship between vaccine beliefs and emotions?

## User Attitudes on Social Media and Vaccination

Expressions of beliefs and emotions about illness on social media invite other users to show their support (Cho et al., 2018). Digital platforms afford users the opportunity to show their attitude toward content posted by others, through clicking the “like” button and commenting. Previous studies have built a framework to evaluate users’ social engagement on Twitter and Facebook, including popularity based on the number of likes, commitments based on the number of comments, and virality based on the number of shares (Bonsón and Ratkai, 2013; Haro-de-Rosario et al., 2018). They have shown that anti-vaccine information with negative emotions was more likely to generate engagement which fosters greater vaccine hesitancy (Keelan et al., 2007; Ache and Wallace, 2008; Briones et al., 2012). However, very few studies had explored the emotional effects of content characteristics on content engagement (Schreiner et al., 2021).

Commenting is regarded as a deeper level of engagement (Cho et al., 2018). Comments not only show audiences’ reactions to the video but also form an overall opinion climate on social media and have the ability to sway users’ prior attitudes (Shi et al., 2021). This suggests that the way people respond to the content could shape the views of other people and thus should be investigated (Sung and Lee, 2015). Therefore, apart from the number of comments, the attitudes portrayed by comments are important indicators to measure vaccine hesitancy, which needs to be explored. In this study, we decided to use the number of comments that supported the argument in the video, as a measure of support on TikTok. We therefore formulated the RQ4:

RQ4: What type of emotions can predict the number of comments that support a video’s argument?

When examining the content of vaccine-related beliefs and user interactions, most existing literature agreed that anti-vaccine content generated more influence than pro-vaccine content (Covolo et al., 2017; Moon and Lee, 2020). However, no research has yet examined the effect of beliefs expressed by pro-vaccine content on social media. Therefore, the final RQ is as follows:

RQ5: What type of beliefs can predict the number of supportive comments?

## Materials and Methods

This study analyzed pro-vaccine videos on the short-video platform TikTok. Two hundred trending videos with the most likes that used the hashtag #vaccine on December 23, 2021 were identified, which reflects the latest trend of vaccine content on this platform. #Vaccine was chosen because it had the highest number of views (6.6 billion) on the platform compared with other vaccine-related hashtags like #covidvaccine (2.3 billion), at the time of data collection. The videos were extracted by using Selenium and the Python programming languages. ChromeDriver, a standalone server, was used to collect videos, which allowed us to run Selenium test scripts on the Google Chrome browser in incognito mode to avoid systematic recommendation algorithms based on history and cookies. The study included the videos directly related to COVID-19 vaccination and excluded videos on other types of vaccination, other topics, or in languages other than English (*n* = 29), as well as videos that were against vaccination (*n* = 12). Moreover, we collected the metadata of each video (including posting date), number of views, number of likes, and 200 comments with the most likes of each video. All data used by this study were posted publicly and identifiable information was removed.

### Coding Scheme

The unit of analysis was a video. The key variables for the content analysis were the beliefs and emotions expressed in the video. Based on a preliminary review of the literature, we used the 5C model to identify the beliefs toward vaccines in videos. It used five main topics (confidence, constraints, complacency, calculation, and collective responsibility) to measure people’s vaccine beliefs (Table 1).

**TABLE 1:** Vaccine beliefs expressed in TikTok videos.

Topic	Sub-topic	Explanation
Complacency	Necessity	Video talks about the necessity of COVID-19 vaccinations
	Importance	Video talks about the importance of COVID-19 vaccinations
Confidence	Effectiveness	Video talks about the effectiveness of COVID-19 vaccinations
	Safety	Video talks about the safety of COVID-19 vaccinations
Constraint	Affordability	Video talks about COVID-19 vaccination being free^1^
	Availability	Video talks about the availability of COVID-19 vaccination
Calculation	Risk assessment	Video talks about both benefits and side effects of the vaccination
	Deliberation	Video provides information that helps improve users’ understanding of the vaccination
Collective responsibility	Concern for others	Video talks about how getting vaccinated helps to protect others or show empathy to them

*^1^Affordability originally depicted the belief that vaccination was affordable, but it was adjusted to measure content that stressed that COVID-19 vaccination was free because the COVID-19 vaccine was provided by governments globally without any charges.*

Each topic was coded as a separate binary (yes/no) variable. If it was mentioned in the video, “1” was assigned; otherwise, “0.” For instance, if a video said that getting vaccinated is safe and very important to stop the spread of the virus, then “1” was assigned to “importance” and “safety,” as well as “complacency” and “confidence.”

Unlike other vaccines, the COVID-19 vaccine is free in the United States and in many other countries; therefore, we decided that the affordability aspect should be removed because few would emphasize common sense to persuade others to be vaccinated (but we still coded this item and the result supported this hypothesis, as only 4% of all the gathered videos stressed that the vaccine was free). There were only eight sub-topics after deleting affordability.

For vaccine emotion, we followed previous empirical studies on emotions (Geronikolou et al., 2021), assessing the presence and absence of the six “basic emotions” proposed by psychologist Ekman (1992). Based on the communication functions of facial behaviors, Ekman showed that facial expressions of six basic emotions (anger, fear, disgust, happiness, sadness, and surprise) could be recognized across different cultures (Ekman and Friesen, 1971), which suited our investigation on a digital platform with global users. Video usually carries a variety of emotions and is effective at evoking them. Thus, emotions were coded as non-mutually exclusive categorical variables.

Anger represented a strong, uncomfortable response to anti-vaccination arguments. If a video expressed anger toward people who refused to be vaccinated or an argument made by anti-vaxxers, then “1” was assigned to “anger.” Happiness represented the joy of being vaccinated. If a video showed how someone enjoyed the process of being vaccinated or made fun of some so-called “side effects,” which were invented by anti-vaxxers, then “1” was assigned to “happiness.” Surprise was a depiction of unexpected emotion. If a video showed that someone was surprised by the arguments of anti-vaxxers, then “1” was assigned to “surprise.” Disgust was an emotional response of rejection or revulsion. If a video expressed a strong rejection or revulsion toward an anti-vaccine argument, then “1” was assigned to “disgust.” Sadness was a representation of the feeling of loss, grief, and sorrow. If a video showed those feelings, then “1” was assigned to “sadness.” Fear depicted an emotion when facing danger or threat. If a video expressed a strong fear or worry, then “1” was assigned to “fear.”

Apart from video content, we also collected the top 200 comments of each video (*N* = 40,000). After excluding the comments of videos on other types of vaccination, other topics, in other languages, and commercial advertisements, 31,800 comments remained. The attitudes expressed in these comments were also coded. If a video showed support for the video’s argument, then “3” was assigned. If a comment made a counterargument, then “1” was assigned. If a comment did not display support or disagreement, then “2” was assigned.

### Intercoder Reliability

To establish intercoder readability, 20 videos were randomly selected, and two coders coded the types of beliefs and emotions of the content. The agreement of the coded data was high (κ = 0.80). Thereafter, 200 video comments were selected, and four coders coded the attitudes of the posts. The agreement of the coded data was excellent (κ = 0.83).

## Results

### Beliefs and Emotions Characteristics

RQ1 concerned the beliefs that are expressed in #vaccine videos on TikTok. Confidence was the most frequently expressed belief (100/159, 62.9%), followed by constraint (68/159, 42.8%), complacency (42/159, 26.4%), and calculation (42/159, 26.4%), respectively. Collective responsibility was the least expressed belief (19/159, 11.9%). With regard to complacency, necessity was the most frequently expressed subtopic (35/159, 22.0%), followed by importance (22/159, 13.8%). With reference to confidence, belief of vaccine safety was the most frequently expressed subtopic (66/159, 41.5%), followed by effectiveness (53/159, 33.3%). Apropos calculation, expressions regarding the frequency of risk assessment (24/159, 15.1%), and deliberation (21/159, 13.2%) sub-topics were similar.

RQ2 concerned the emotions that are expressed in #vaccine videos on TikTok. Joy was the most frequently expressed emotion (81/159, 50.9%), such as expressing enthusiasm for being vaccinated and mocking so-called side effects of the vaccine, followed by surprise (59/159, 37.1%), such as being shocked by the effects of the vaccine or the attitudes toward the vaccine that other people expressed. These were followed by similar expressions of anger (38/159, 23.9%), such as outrage at vaccine misinformation, and disgust (37/159, 23.3%), such as sarcastic comments about or imitations of vaccine misinformation and misbehaviors. Fear, such as worry about the effectiveness of vaccines, ranked fifth (17/159, 10.7%). Sadness was rarely expressed (7/159, 4.4%).

RQ3 concerned the relationship between beliefs and emotions. Chi-square tests of independence were performed to determine whether emotions are contingent on beliefs. The results indicated that joy was correlated with complacency, χ^2^ (1, *n* = 159) = 5.297, *P* = 0.017; constraint, χ^2^ (1, *n* = 159) = 5.567, *P* = 0.014; and calculation χ^2^ (1, *n* = 159) = 7.083, *P* = 0.006. Surprise was correlated with confidence, χ^2^ (1, *n* = 159) = 7.194, *P* = 0.005. Fear was also correlated with confidence, χ^2^ (1, *n* = 159) = 7.952, *P* = 0.003. Disgust was correlated with complacency, χ^2^ (1, *n* = 159) = 7.025, *P* = 0.009; constraint, χ^2^ (1, *n* = 159) = 4.881, *P* = 0.020; and collective responsibility, χ^2^ (1, *n* = 159) = 7.018, *P* = 0.012. Anger was correlated with complacency, χ^2^ (1, *n* = 159) = 8.624, *P* = 0.004; calculation, χ^2^ (1, *n* = 159) = 6.324, *P* = 0.012; and collective responsibility, χ^2^ (1, *n* = 159) = 6.535, *P* = 0.015. Since few videos expressed sadness, the chi-square test model of sadness was non-significant. Table 2 presents the correlations between emotions and vaccine beliefs.

**TABLE 2:** The chi-square (χ^2^) test of emotions and vaccine beliefs.

	Complacency		Confidence		Constraint		Calculation		Collective responsibility	
	*n* (%)	χ^2^	*n* (%)	χ^2^	*n* (%)	χ^2^	*n* (%)	χ^2^	*n* (%)	χ^2^
Joy	15 (9.4)	5.297*	49 (30.8)	0.407	42 (26.4)	5.567*	14 (8.8)	7.083**	11 (6.9)	0.417
Surprise	20 (12.6)	2.703	45 (28.3)	7.194**	26 (16.4)	0.065	19 (11.9)	1.617	9 (5.7)	0.974
Sadness	3 (1.9)	1.018	4 (2.5)	0.104	2 (1.3)	0.603	2 (1.3)	0.018	2 (1.3)	1.923
Fear	5 (3.1)	0.088	16 (10.1)	7.952**	7 (4.4)	0.02	5 (3.1)	0.088	0 (0)	2.583
Disgust	16 (10.1)	7.025**	25 (15.7)	0.451	10 (6.3)	4.881*	14 (8.8)	3.237	9 (5.7)	7.018*
Anger	17 (10.7)	8.624**	27 (17.0)	1.425	16 (10.1)	0.009	16 (10.1)	6.324*	9 (5.7)	6.535*

*p < 0.05, **p < 0.01.

### Effects of Beliefs and Emotions Characteristics

RQ4 concerned the type of emotions that can predict the number of comments that support the video’s argument. RQ5 concerned the type of vaccine belief that can predict the number of comments that support the video’s argument.

The average number of comments that supported the video’s argument was 130.89, whereas the average number of comments that did not support the video’s argument was 60.85. There were 1,230 comments whose attitudes could not be identified owing to being in a language other than English, incomplete expression, or unrelated messages.

A multiple regression analysis was performed to predict the number of comments that would support the video’s argument. Five vaccine beliefs (complacency, confidence, constraint, calculation, and collective responsibility) and six emotions (joy, surprise, sadness, fear, disgust, and anger) were entered into the model. The model was significant, *R*^2^ = 0.18, *F*(11,137) = 2.739, *P* < 0.01. Among the five beliefs, only confidence (*B* = 14.84, *P* < 0.05) predicted the number of comments that supported the video. Regarding emotions, surprise (*B* = 11.29, *P* < 0.05) and sadness (*B* = 37.49, *P* < 0.01) predicted the number of comments that supported the video. Table 3 presents the effects of vaccine beliefs and emotions on the number of comments that support the video.

**TABLE 3:** Effects of beliefs and emotions on the number of comments that supports the video.

	Estimate (SE)	*P*
Complacency	−7.82 (−0.10)	0.216
Confidence	14.84 (0.21)	0.013
Constraint	−1.63 (−0.02)	0.773
Calculation	9.47 (0.13)	0.131
Collective responsibility	8.16 (0.08)	0.357
Joy	7.96 (0.12)	0.208
Surprise	11.29 (0.16)	0.048
Sadness	37.49 (0.22)	0.006
Fear	7.72 (0.07)	0.419
Disgust	9.30 (0.12)	0.197
Anger	2.12 (0.03)	0.770

*R^2^ = 0.18, F(11,137) = 2.739.*

## Discussion

The present study identified beliefs and emotions expressed in videos that support COVID-19 vaccines on TikTok and their influences on users’ attitudes toward the vaccines. We found that confidence was the most frequently expressed belief on TikTok and was positively related to the number of comments that supported the video. The results are consistent with several prior findings, which demonstrate that the lack of trust in vaccines leads to vaccine hesitancy (Guay et al., 2019; Roozenbeek et al., 2020; Lindholt et al., 2021). Constraint was the second-most-mentioned-vaccine-related belief in the sample. Although the regression analysis indicated a non-significant relationship between this belief and supportive comments, the estimated coefficient suggests that it may trigger more disagreement on social media. It seems that pro-vaccine arguments stressing the availability of vaccines may not persuade others to show support for them. It is a similar situation for complacency; in particular, for videos that argue vaccination is important and necessary. Although several studies have found that collective responsibility is strongly related to vaccination intention (Lindholt et al., 2021; Wismans et al., 2021), we did not find the same association in the case of TikTok in our study.

Regarding emotions, although joy was the most frequently expressed emotion of videos that supported COVID-19 vaccination, it did not predict the number of comments that supported the video. Sadness was the least-mentioned emotion in the top 200 trending #vaccine videos. However, it significantly increased support on social media, which accords with previous studies of anti-vaccine social media content that found negative emotions generated more user engagements (Keelan et al., 2007; Ache and Wallace, 2008). Surprise was another emotion that predicted the number of comments that supported the video, which is aligned with a study on TikTok advertisements (Hutchinson, 2020). It showed that advertisements emphasizing strong emotions, like surprise, led to a higher view rate. Further, surprise was related to confidence. Pro-vaccine videos that emphasized the effectiveness and safety of vaccines are more likely to show surprise, such as being surprised by the comforting experience of being vaccinated or absurd anti-vaccine arguments.

### Theoretical Implications

This study is the first to apply the 5C model to analyze information. It provides theoretical implications for expanding the application of the 5C framework to social media and visual contents. Our findings provide greater clarity on the influence of specific types of beliefs and emotions, and can be used to further explore the role of social media and user psychology in overcoming vaccine-hesitant behaviors.

We argue that social media content promoting the effectiveness and safety of the COVID-19 vaccine is the most helpful in overcoming vaccine hesitancy. According to the TRA, individuals behave based on their behavioral intentions, which are determined by their attitudes and subjective norms. Videos expressing confidence on the COVID-19 vaccination contribute to the enhancement of perceived usefulness, which is proved to have a positive impact on vaccine acceptance (Akther and Nur, 2022). Additionally, a previous study showed that opinions from the social environment can predict users’ acceptance of vaccines (Baldwin et al., 2013). Since social media serves not only as an information source but also as an important social environment for individual users, beliefs regarding the COVID-19 vaccine that could exert effects on attitudes would have an impact on vaccine intentions and behaviors and help combat misinformation on social media.

Furthermore, this study offered a detailed analysis on specific types of emotions and their effects, providing a good cutting point to discuss the role of social media and user psychology in addressing vaccine hesitancy. The uses and gratifications theory (Katz et al., 1974) suggests that the needs, motives, and gratifications of media users influence media consumptions. One recent study suggests that the gratification of entertainment and affective needs are the most primary drivers when consuming video content on TikTok (Bossen and Kottasz, 2020). By evoking sadness and surprise emotions, pro-vaccine videos are more likely to attract attention and gain support. It is possible that the surprise emotion fulfills users’ entertainment needs while sadness can fulfill their affective needs resultant from feelings of depression and loss in the pandemic. Following studies are needed to establish more solid relations among content emotions, user gratifications, and vaccine hesitancy.

### Practical Implications

In addition to extending theoretical understanding about vaccine hesitancy, our study helps form better strategies in promoting COVID-19 vaccines, and consequently, improve vaccine uptake during the pandemic. First, emphasizing the safety and effectiveness of vaccination is the most helpful to promote vaccines. For health departments and other related institutions, it is important to place emphasis on enhancing confidence to elicit support on social media to promote vaccines. Second, considering the fact that complacency is negatively associated with vaccination intention (Wismans et al., 2021), we should consider stressing more information beyond the importance, necessity, and availability of vaccines when promoting COVID-19 vaccines in the future. Third, emotional expressions, especially sadness and surprise, should be an important part of future vaccine promotion campaigns. Since synthesis of promotion strategies was more effective in increasing vaccination coverage (Frew and Lutz, 2017), pro-vaccine videos emphasizing confidence in vaccines, together with surprise, would be a good combination for short-video promotion.

### Limitations and Future Research

The current study has some limitations. First, due to the sampling procedure, anti-vaccine videos and videos without #vaccine were not included. Further studies that test the relationship between beliefs and emotions expressed in anti-vaccine and neutral contents on social media can compare their effects with this study. Second, as the first investigation into beliefs, emotions, and comments on TikTok relating to COVID-19 vaccination, this study does not consider the number of views, likes, and comments owing to inadequate knowledge of the recommendation algorithm. However, the algorithm plays an important role in information dissemination and in shaping individual user attitudes and overall online opinion by favoring the emergence of echo chambers and filter bubbles (Colleoni et al., 2014; Schmidt et al., 2018). Algorithms influence how people with different attitudes toward vaccines encounter each other. Subsequent studies should examine how algorithms influence the interaction between social media users with different vaccine attitudes.

## Conclusion

This study examined trending TikTok videos and comments, and offered insights into what types of vaccine beliefs and emotions on social media can help address COVID-19 vaccine hesitancy. The findings show that confidence is the most frequently expressed belief and is positively related to the supporting comments, which is consistent with similar studies. Sadness, when compared to other negative emotions including fear, anger, and disgust, is more likely to gain support when popularizing COVID-19 vaccines. In addition, surprise is another emotion that predicted the number of support. The results suggest that emotional expressions should be an important part of future vaccine promotion campaigns. Promoting short-videos using synthesis of promotion strategies, like emphasizing confidence in vaccines with the surprise emotion, would be a more effective approach to develop vaccine confidence.

## Data Availability Statement

The original contributions presented in this study are included in the article/supplementary material, further inquiries can be directed to the corresponding author.

## Author Contributions

XW and RH contributed to the conception and design of the study. XW organized the database and coding process. RH performed the statistical analysis. Both authors wrote the first draft of the manuscript, contributed to manuscript revision, and read and approved the submitted version.

## Conflict of Interest

The authors declare that the research was conducted in the absence of any commercial or financial relationships that could be construed as a potential conflict of interest.

## Publisher’s Note

All claims expressed in this article are solely those of the authors and do not necessarily represent those of their affiliated organizations, or those of the publisher, the editors and the reviewers. Any product that may be evaluated in this article, or claim that may be made by its manufacturer, is not guaranteed or endorsed by the publisher.
